# Gastrodin Attenuates Cerebral Ischemia–Reperfusion Injury by Enhancing Mitochondrial Fusion and Activating the AMPK‐OPA1 Signaling Pathway

**DOI:** 10.1111/cns.70559

**Published:** 2025-08-11

**Authors:** Zihan Liu, Zeyu Han, Wenshuai Bao, Yihan Guo, Yuan Yuan, Jianming Cheng, Jie Zhang, Yang Hu

**Affiliations:** ^1^ Jiangsu Province Engineering Research Center of Classical Prescription, School of Pharmacy Nanjing University of Chinese Medicine Nanjing China; ^2^ Xian Jiaotong‐Liverpool University XJTLU Wisdom Lake Academy of Pharmacy Suzhou China; ^3^ School of Traditional Chinese Medicines China Pharmaceutical University Nanjing China

**Keywords:** AMPK‐OPA1, cerebral ischemia–reperfusion injury, Gastrodin, mitochondrial fusion, oxygen–glucose deprivation/reperfusion

## Abstract

**Background:**

Cerebral ischemia–reperfusion (I/R) injury is a critical pathological process in stroke, characterized by disrupted energy metabolism, inflammatory responses, and mitochondrial dysfunction. Targeting mitochondrial dynamics presents promising strategies for alleviating brain injury. This study investigates the role and mechanism of Gastrodin (Gas) in regulating mitochondrial dynamics and mitigating cerebral I/R injury via activation of the AMPK‐OPA1 signaling pathway.

**Methods:**

An in vitro oxygen–glucose deprivation/reperfusion (OGD/R) model and an in vivo middle cerebral artery occlusion/reperfusion (MCAO/R) model were used to assess the effects of Gas on inflammation, mitochondrial function, and energy metabolism. Immunofluorescence, western blotting (WB), reverse‐transcription PCR (RT‐PCR), JC‐1 staining, and molecular docking techniques were employed for analysis.

**Results:**

Gas activated the AMPK‐OPA1 signaling pathway, promoting mitochondrial fusion, restoring membrane potential, enhancing ATP production, and rebalancing NAD^+^/NADH levels. Additionally, Gas significantly suppressed I/R‐induced inflammatory responses, reduced neuronal damage, and decreased infarct volume. Notably, its protective effects on mitochondrial fusion and neuroprotection were abolished under AMPK silencing, highlighting the critical role of the AMPK‐OPA1 pathway.

**Conclusion:**

Gas alleviates cerebral I/R injury by regulating mitochondrial dynamics via the AMPK‐OPA1 signaling pathway. These findings provide a theoretical basis for the therapeutic application of Gas in stroke and offer new insights into mitochondrial‐targeted treatment strategies.

## Graphical abstract

Gastrodin (Gas) exerts neuroprotective effects in mitigating cerebral I/R injury predominantly through activation of the AMPK‐OPA1 signaling pathway to regulate mitochondrial dynamics.

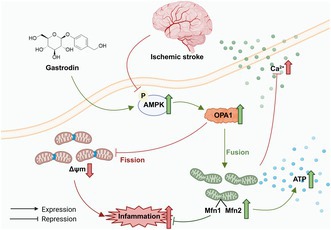

## 1 Introduction

Stroke, also known as a cerebrovascular accident, is a condition characterized by impaired cerebral blood flow, leading to structural and functional damage to brain tissue due to vessels blockage or rupture [1]. Strokes are classified as ischemic stroke (IS) or intracerebral hemorrhage (ICH) strokes, and about 87% of strokes are ischemic [2, 3]. The pathological basis of IS is the reduction of blood flow to brain tissue caused by arterial occlusion. The deprivation of oxygen and nutrients disturbs cellular homeostasis and ultimately leads to ischemic death of neuronal cells [4].

Neurons require substantial energy to maintain their function and homeostasis, with mitochondria acting as the primary energy producing organelles. Mitochondria generate cellular energy in the form of ATP through oxidative phosphorylation, a process facilitated by the mitochondrial electron transport chain (ETC). In the context of ischemic brain injury, the diminished supply of oxygen and glucose critically disrupts mitochondrial oxidative phosphorylation, leading to a marked reduction in ATP production and a breakdown of cellular energy metabolism [5]. This energy deficit not only compromises cellular function but also heightens susceptibility to oxidative stress and calcium overload, ultimately accelerating neuronal death.

The mitochondrial network exhibits high dynamism, undergoing continuous cycles of fusion and fission to respond to metabolic or environmental stresses, thereby regulating the balance of mitochondrial morphology, distribution, and function [6]. During stroke, oxidative stress leads to the hyperactivation of dynamin‐related protein 1 (Drp1), a key protein involved in mitochondrial fission, resulting in excessive mitochondrial fragmentation [7, 8]. Such fragmentation disrupts mitochondrial integrity and contributes to neuronal damage. Conversely, mitochondrial fusion plays a protective role by integrating damaged or dysfunctional mitochondria into a healthier network [9]. This process facilitates inter‐mitochondrial communication, promotes repair and functional recovery, and prevents further mitochondrial fragmentation [10]. Optic atrophy 1 (OPA1), a dynamin superfamily GTPase involved in membrane remodeling, mediates the fusion of inner mitochondrial membranes, enhancing oxidative phosphorylation (OXPHOS), a critical process for mitochondrial energy metabolism [11, 12]. 5′‐AMP‐activated protein kinase (AMPK), a key serine/threonine kinase, is ubiquitously expressed in various cells and tissues. As a primary cellular energy sensor, AMPK is activated under conditions of low energy [13]. It functions by phosphorylating specific enzymes and regulatory proteins to enhance ATP production and suppress ATP consumption. Recent studies underscore the pivotal role of AMPK in maintaining mitochondrial homeostasis through its regulation of mitochondrial biogenesis, dynamics, and quality control. AMPK promotes mitochondrial biogenesis to regulate mitochondrial quantity, modulates mitochondrial morphology to maintain network stability, and ensures mitochondrial quality by facilitating autophagy and mitophagy [14]. Collectively, these functions highlight the essential role of AMPK in sustaining energy metabolism and preserving cellular homeostasis, particularly under stress conditions such as stroke [15, 16].

Gastrodiae Rhizoma (*Gastrodia elata* Bl.), the dried tuber from the Orchidaceae family [17], has been widely recognized in traditional Chinese medicine for its neuroprotective properties [18, 19, 20, 21], with its primary bioactive component, Gas, demonstrating efficacy through multiple mechanisms. Gas exerts antioxidant effects by scavenging reactive oxygen species (ROS), thereby reducing oxidative stress, and it mitigates neuroinflammation by suppressing the expression of pro‐inflammatory cytokines such as IL‐1β and TNF‐α, as well as downregulating key inflammatory signaling pathways [22]. Furthermore, it inhibits neuronal apoptosis by modulating the Bcl‐2/Bax ratio and preventing caspase‐3 activation [23, 24]. Gas also promotes angiogenesis and neural repair through the activation of the Wnt/β‐Catenin signaling pathway [25], enhancing vascular endothelial growth factor (VEGF) expression [24], and facilitating the proliferation and differentiation of neural stem cells. Additionally, it alleviates pyroptosis by inhibiting the activation of the NLRP3 inflammasome [26], further protecting against cerebral tissue damage. However, no studies have been conducted to further explore its mechanism of action from the perspective of regulating mitochondrial homeostasis.

Building on the aforementioned findings, we hypothesize that Gas exerts neuroprotective effects by activating the AMPK‐OPA1 signaling pathway, thereby mitigating mitochondrial fragmentation and preventing neuronal damage associated with ischemia–reperfusion injury. To validate this hypothesis, we utilized both the OGD/R model in SH‐SY5Y cells and the MCAO/R rat model to explore the mechanisms by which Gas regulates mitochondrial dynamics in the context of stroke treatment. This study provides further insights into the pathophysiological mechanisms of stroke and introduces a potential therapeutic strategy for neuroprotection.

## 2 Materials and Methods

### 2.1 Reagents

All reagents were obtained from Beyotime (China) unless stated otherwise. AMPK, p‐AMPK, β‐actin, and Tom20 monoclonal Abs were obtained from Proteintech (China). Mitofusin 1 (Mfn1) and mitofusin 2 (Mfn2) monoclonal Abs were purchased from ABclonal (China). GFAP monoclonal Ab was from Huabio (China). AMPK siRNAs were purchased from Tsingke (China). DAPI and all fluorescent‐labeled secondary antibodies were purchased from Abbkine (China). HRP‐conjugated goat‐anti‐mouse and goat‐anti‐rabbit secondary antibodies were from Proteintech (China).

### 2.2 Cell Culture, OGD/R, and Drug Treatment

Human neuroblastoma cells (SH‐SY5Y) were obtained from Shanghai Institute of Cell Biology, Chinese Academy of Sciences. SH‐SY5Y cells were cultured in Dulbecco's Modified Eagle's Medium (DMEM, keygenbio, China) supplemented with 10% fetal bovine serum (FBS, ExCell Bio, China) and routinely cultured at 37°C with 5% CO_2_. An in vitro cerebral ischemia–reperfusion model was established using the OGD/R method. During OGD/R, cells were initially cultured for 12 h in serum‐ and glucose‐free DMEM medium (Biosharp, China) or gas‐containing serum‐ and glucose‐free DMEM medium. Subsequently, the cells were transferred to a hypoxic environment (94% N_2_, 1% O_2_, 5% CO_2_) for 12 h to simulate ischemic conditions. At the time of reperfusion, the medium was replaced with normal culture medium, and cells were cultured for an additional 24 h before proceeding with subsequent experiments.

### 2.3 Western Blot

SDS‐PAGE electrophoresis was used to separate proteins of different molecular weights. After electrophoresis, the proteins on the gel were transferred to a NC membrane. The following samples were sealed with 5% BSA or 5% skim milk powder for 2 h, and then incubated at 4°C for the entire night: AMPK (1:500), p‐AMPK (1:1000), OPA1 (1:1000), Mfn1 (1:1000), Mfn2 (1:1000)、GFAP (1:1000) and β‐actin (1:5000). After an overnight wash with TBS‐T three times for 15 min each, secondary antibodies (1:5000) were incubated for 2 h at room temperature. The bands were detected by the ECL kit, and then they were quantified using ImageJ software V1.8.0 (Bethesda, MD, USA).

### 2.4 RT‐PCR

Total RNA was isolated from treated macrophages using TRIzol reagent and reverse transcribed to cDNA using a cDNA synthesis kit (Applied Biological Materials, Canada) according to the manufacturer's protocol. RT‐PCR was then performed using SYBR Green Mastermix, according to the manufacturer's instructions, and run on a LightCycler 480II real‐time fluorescence quantitative PCR instrument. Data was analyzed using the 2^−ΔΔCT^ method. Primer sequences for RT‐PCR analysis are shown in the Supporting Information.

### 2.5 Immunofluorescent Analysis

For visualization of mitochondria morphology, the cultivated cells underwent three PBS washes before being fixed for half an hour at room temperature, treated for 20 min with 0.5% Triton X‐100, and blocked for 60 min with 10% normal goat serum. Rabbit monoclonal antibodies against Tom20 (1:500) were applied to the cells overnight at 4°C after they had been sealed. After three PBS washes, the cells were treated with the secondary antibody and incubated for 1 h at room temperature with FITC goat‐anti‐rabbit secondary antibody (1:500), shielded from light. Following incubation, the cells were gently washed three times in PBS before being restained with DAPI for 5 min. Following that, a confocal laser scanning microscope was used to take pictures of the cells.

### 2.6 Cellular Thermal Shift Assay (CETSA)‐Western Blotting Assays

CETSA‐Western blotting, which integrates the CETSA with Western blotting. In essence, SH‐SY5Y cells were exposed to Gas at a concentration of 25 μM or an equivalent volume of DMSO for 1 h under ambient temperature. The lysates were subjected to heat treatment within 32°C–77°C for 3 min. Next, the lysates were cooled to 4°C for another 3 min. Following thermal cycling, the soluble protein fraction was isolated by centrifugation at 20,000× *g* for 20 min at 4°C. The liquid portion with a relatively high concentration of soluble proteins, known as the supernatant, was collected for examination via Western blot analysis.

### 2.7 Transfection

SH‐SY5Y cells were transfected with AMPKα1‐specific small interfering RNA (siRNA) sequence according to the manufacturer's instructions as follows: si‐AMPKα1#1 5′‐GCAGAAGUAUGUAGAGCAAUC‐3′; siAMPKα1#2 5′‐GCUUGAUGCACACAUGAAU‐3′; si‐AMPKα1#3 5′‐CCUUUCUGGUGUGGAUUAU‐3′. After 48 h of siRNA transfection, the protein expression of AMPK was evaluated by WB and Image J.

### 2.8 JC‐1 and Fluo‐4 Staining

The mitochondrial membrane potential was tested by a JC‐1 probe according to the manufacturer's protocol. SH‐SY5Y cells were incubated with 10 mg/mL JC‐1 for 10 min at 37°C in the dark and monitored with a fluorescence microscope and flow cytometer. The red‐orange fluorescence reflects the potential‐dependent dye aggregation in the mitochondria, and the green fluorescence represents the monomeric form of JC‐1, which suggests the depolarization of the mitochondrial membrane. Intracellular calcium concentrations were assessed using the Fluo‐4 calcium ion detection kit. SH‐SY5Y cells were washed with PBS and subsequently incubated with Fluo‐4 staining solution at 37°C in the dark for 30 min. After incubation, cells were washed once with PBS, followed by Hoechst 33258 staining at room temperature for 10 min. The cells were then washed three times with PBS and observed under a fluorescence microscope for analysis.

### 2.9 MCAO/R Model and Drug Treatment

Adult male Sprague Dawley (SD) rats (body weight, 240–260 g) were purchased from Jiangsu Qinglongshan Biotechnology Co. Ltd. All experiments were in complete compliance with the National Institutes of Health Guide for the Care and Use of Laboratory Animals and were approved by the Animal Care and Use Committee of Nanjing University of Chinese Medicine (no. 20220112A, 20240315A). The preparation of the MCAO/R model is briefly described as follows: Prior to surgery, a nylon thread was disinfected with 75% alcohol and marked 18.5 mm from the tip using a waterproof black marker, then stored in sterile 0.9% saline. Rats were anesthetized with Schutai Z50 (50 mL/kg, ip) and placed in a supine position on the surgical table. A midline neck incision was made to expose the left common carotid artery (CCA), external carotid artery (ECA), internal carotid artery (ICA), and the branches of the ECA. The ECA was ligated distally, and the CCA was ligated proximally. An arterial clamp was applied to occlude both the CCA and ICA. A 2/3 diameter incision was made in the ECA, through which the prepared nylon thread was inserted into the ICA to occlude blood flow, and secured with a surgical knot. After 2 h of MCA occlusion, the nylon thread was removed from the ACA, and reperfusion was initiated by restoring blood flow through the ECA, with reperfusion continuing for 22 h. This model effectively simulates cerebral ischemia/reperfusion injury in rats for subsequent evaluation of therapeutic interventions.

### 2.10 Neurological Score

Neurological function in rats was evaluated 24 h after cerebral ischemia–reperfusion using the Longa scoring system, which includes five grades: 0 points for no neurological deficits and normal function; 1 point for mild impairment, where the left forelimb cannot fully extend; 2 points for moderate impairment, where the animal circles towards the paralyzed side during walking; 3 points for severe impairment, where the animal tilts towards the paralyzed side while walking; and 4 points for the most severe impairment, where the animal is unable to walk independently and shows loss of consciousness.

### 2.11 2, 3, 5‐Triphenyltetrazolium Chloride (TTC) Staining

For TTC staining, rats were anesthetized and their brains were rapidly removed while maintaining tissue integrity. The excised brain was immediately frozen at −20°C for approximately 20 min. Following freezing, the brain was sectioned into 5–6 slices, each approximately 2 mm thick. The brain slices were then placed in a 2% TTC solution in a 24‐well plate and incubated in a 37°C incubator for 15 min in the dark. During this incubation, the slices were gently flipped to ensure uniform staining. Normal tissue stained red, while infarcted tissue remained pale. After staining, the slices were fixed in 4% paraformaldehyde for 24 h. The fixed slices were photographed using a digital camera, and the infarct area was calculated. The ischemic area was quantified by calculating the ratio of the infarcted area (white regions) to the total area of each slice.

### 2.12 Hematoxylin and Eosin (HE) Staining

For HE staining, fixed brain tissues were processed through a standard dehydration protocol using a graded ethanol series, followed by xylene clearing and paraffin embedding. Tissue sections were cut using a microtome and mounted on glass slides, then air‐dried. The slides were stained with hematoxylin and eosin, followed by dehydration with ethanol and clearing in xylene. Finally, the sections were mounted with a coverslip. Brain tissue was scanned using a Leica digital slide scanner, and specific brain regions were analyzed using ImageScope software for detailed evaluation.

### 2.13 Nissl Staining of Brain Tissue

Brain tissue sections were first hydrated by placing them in pure water for 3–5 min, followed by staining with tar purple solution for 1–1.5 h. After staining, the sections were rinsed in pure water to remove excess dye. Differentiation was achieved by sequential immersion in 70%, 80%, and 95% ethanol for 5–8 s each, ensuring the preservation of staining intensity. The sections were then treated with a special differentiating solution (1:1:1 anhydrous ethanol, chloroform, and ether) to eliminate background staining. After dehydration in 100% ethanol (5 min × 3), the sections were cleared with xylene (5 min × 3) and mounted for further analysis. Throughout the procedure, care was taken to maintain the sections in a hydrated state. Under light microscopy, Nissl bodies stained deep blue‐purple, while the nuclei and nucleoli appeared pale purple, with a clean, colorless background. Brain tissue scanning and analysis were performed using the Leica digital slide scanner system, and specific brain regions were analyzed using ImageScope software.

### 2.14 Statistical Analysis

The results were analyzed using Prism 8.0 (GraphPad Software) and provided as mean ± SEM. (*n* ≥ 3). Normality was confirmed using the Shapiro–Wilk test. Comparisons between two groups were conducted with Student's *t*–test; whereas multiple group comparisons were analyzed using one‐way or two‐way analysis of variance (ANOVA). Statistical significance was defined as *p* < 0.05 for all analyses.

## 3 Results

### 3.1 Inhibition of the AMPK‐OPA1 Signaling Pathway Exacerbates Mitochondrial Fragmentation Induced by OGD/R in SH‐SY5Y Cells

**FIGURE 1:**
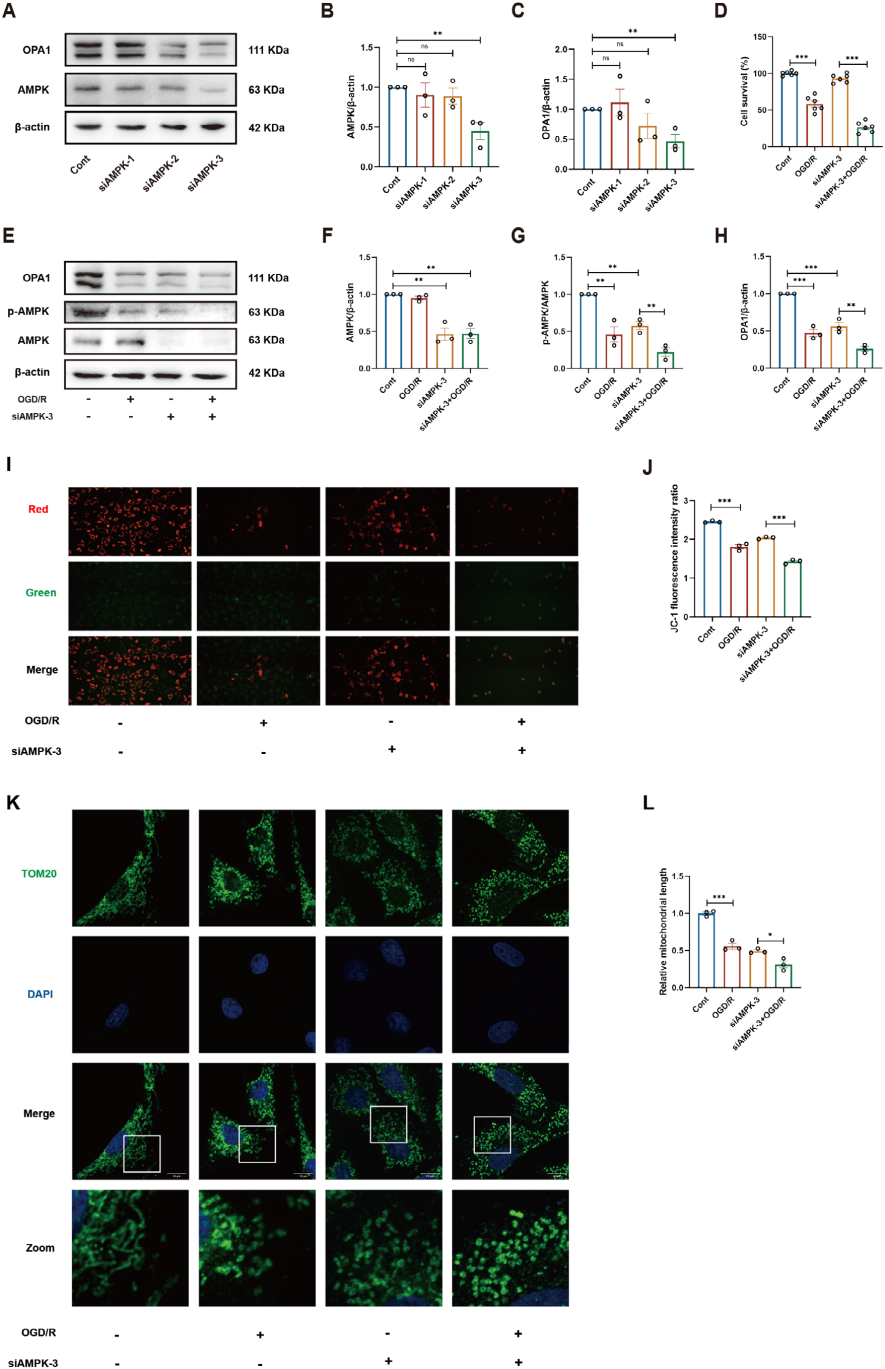
Inhibition of the AMPK‐OPA1 signaling pathway exacerbates mitochondrial fragmentation induced by OGD/R in SH‐SY5Y cells. (A–C) WB analysis of AMPK and OPA1 expression in SH‐SY5Y cells transfected with different AMPK siRNAs (*n* = 3). (D) Cell viability of SH‐SY5Y cells subjected to OGD/R or AMPK silencing, assessed by CCK‐8 assay (*n* = 6). (E–H) WB analysis of AMPK, p‐AMPK, and OPA1 protein levels in SH‐SY5Y cells following OGD/R or AMPK silencing (*n* = 3). (I, J) The JC‐1 assay in SH‐SY5Y cells following OGD/R or AMPK silencing and quantitative analysis of mitochondrial membrane potential (*n* = 3). (K, L) Morphological analysis of mitochondria in SH‐SY5Y cells after OGD/R or AMPK silencing and quantitative analysis of average mitochondrial length (*n* = 3). Data are presented as mean ± SEM; *n* ≥ 3 independent experiments. *p < 0.05, **p < 0.01, ***p < 0.001.

To elucidate the relationship between AMPK and OPA1, we transfected siRNA of AMPK into SH‐SY5Y to demonstrate that AMPK is an upstream mediator of OPA1. We compared three siRNAs for AMPK, and siAMPK‐3 was the most effective (Figure 1A–C). OPA1 was also found to decrease with AMPK silencing. The results of CCK‐8 experiments showed that silencing of AMPK exacerbated OGD/R‐induced cellular damage (Figure 1D). On this basis, we found that OPA1 expression was significantly suppressed in SH‐SY5Y cells after OGD/R by WB experiments, which demonstrated the importance of the AMPK‐OPA1 pathway in the pathogenesis of stroke (Figure 1E–H). Excessive mitochondrial fragmentation is usually accompanied by a decrease in membrane potential [27], and we used a JC‐1 fluorescent probe to determine the mitochondrial membrane potential. As shown in Figure 1I,J, inhibition of the AMPK‐OPA1 pathway exacerbated the OGD/R‐induced decrease in SH‐SY5Y mitochondrial membrane potential. Mitochondrial morphology was observed under confocal microscopy by TOM‐20 labeling of mitochondria to mark the outer mitochondrial membrane (Figure 1K,L). As expected, silencing of AMPK‐OPA1 significantly further exacerbated the OGD/R‐induced fragmentation of mitochondria to form donut‐shaped mitochondria.

### 3.2 Gas Inhibits Inflammatory Response Induced by OGD/R With Elevated Intracellular Ca2^+^ Concentration to Protect SH‐SY5Y Cells

**FIGURE 2:**
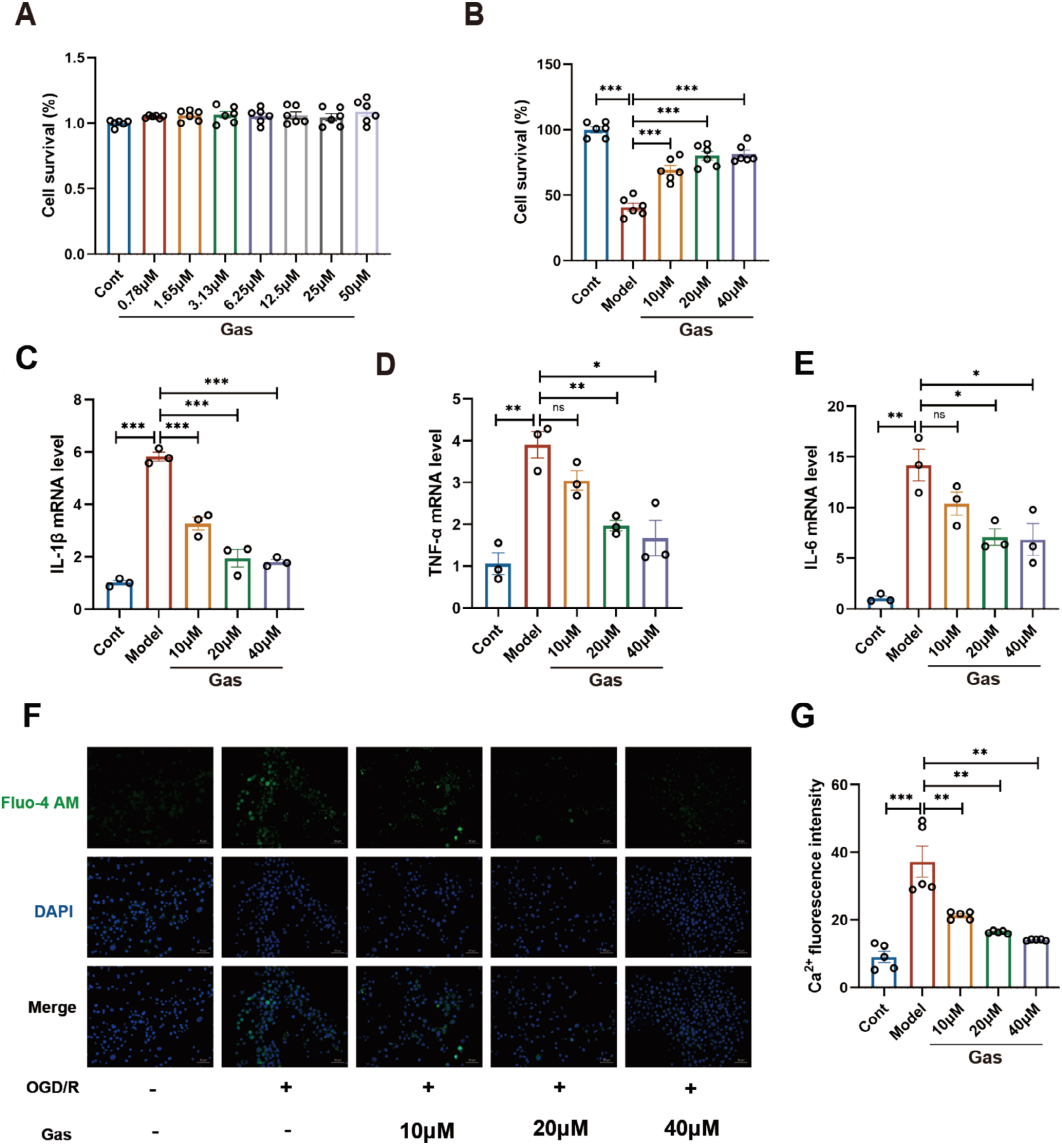
Gas inhibits inflammatory response induced by OGD/R with elevated intracellular Ca^2+^ concentration to protect SH‐SY5Y cells. (A) The cell viability of SH‐SY5Y cells was assessed by CCK‐8 assay after intervention with different concentrations of Gas (*n* = 6). (B) CCK‐8 assay was performed to evaluate the protective effects of Gas on SH‐SY5Y cells subjected to OGD/R (*n* = 6). (C–E) RT‐PCR analysis of mRNA expression levels of pro‐inflammatory cytokines IL‐1β, TNF‐α, and IL‐6 in SH‐SY5Y cells (*n* = 3). (F, G) Intracellular Ca^2+^ overload was monitored and quantified by the fluorescent Ca^2+^ indicator Fluo‐4 AM (*n* = 5). Data are presented as mean ± SEM; *n* ≥ 3 independent experiments. *p < 0.05, **p < 0.01, ***p < 0.001.

At concentrations that do not exhibit cytotoxicity in SH‐SY5Y cells (Figure 2A), Gas was applied to OGD/R‐treated SH‐SY5Y cells at concentrations of 10, 20, and 40 μM. The results demonstrated that Gas conferred neuroprotective effects in a dose‐dependent manner (Figure 2B). Ischemia–reperfusion injury is known to trigger an inflammatory response; thus, RT‐PCR was employed to assess the expression levels of key inflammatory cytokines, including TNF‐α, IL‐6, and IL‐1β, across different treatment groups. The findings revealed that Gas effectively inhibited the expression of these inflammatory mediators in a dose‐dependent fashion (Figure 2C–E). During cerebral ischemia, the interruption of blood flow and consequent energy depletion leads to irreversible necrosis of neurons in the ischemic core. Impaired glucose and energy metabolism reduce Na^+^/K^+^‐ATPase activity, resulting in ionic imbalance and membrane depolarization. This cascade promotes excessive release of the excitatory neurotransmitter glutamate, which binds to glutamate receptors and accelerates Ca^2+^ influx. The resulting mitochondrial dysfunction ultimately induces apoptosis 31. To evaluate changes in intracellular Ca^2+^ concentration, Fluo‐4AM was used as a fluorescent marker (Figure 2F,G). Immunofluorescence analysis indicated that Gas treatment at varying doses effectively alleviated the pathological increase in intracellular Ca^2+^ levels in SH‐SY5Y cells.

### 3.3 Gas Improves Mitochondrial Membrane Potential and Energy Metabolism in SH‐SY5Y Cells After OGD/R

**FIGURE 3:**
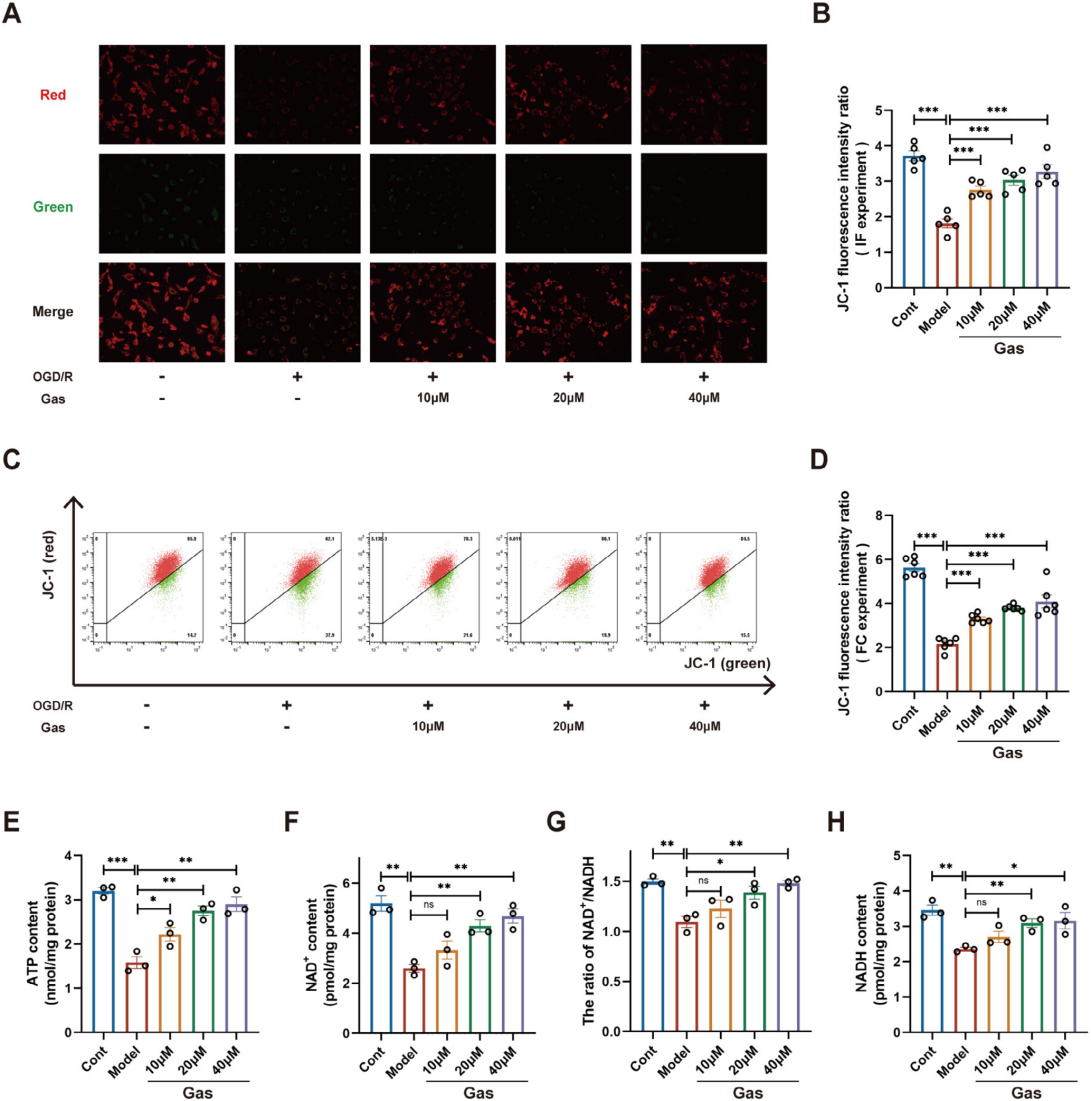
Gas improves mitochondrial membrane potential and energy metabolism in SH‐SY5Y cells after OGD/R. (A, B) Mitochondrial membrane potential in SH‐SY5Y cells from each group was assessed using JC‐1 immunofluorescence staining (*n* = 5). (C, D) Mitochondrial membrane potential was assessed in each group of SH‐SY5Y cells using flow cytometry to detect JC‐1 staining (*n* = 6). (E) ATP levels were quantified in SH‐SY5Y cells (*n* = 3). (F) NAD^+^ levels were quantified in SH‐SY5Y cells (*n* = 3). (G) NAD^+^/NADH levels were quantified in SH‐SY5Y cells (*n* = 3). (H) NADH levels were quantified in SH‐SY5Y cells (*n* = 3). Data are presented as mean ± SEM; *n* ≥ 3 independent experiments. *p < 0.05, **p < 0.01, ***p < 0.001.

The brain is one of the most energy‐demanding organs in the body, accounting for approximately 20% of the basal metabolic rate. Its normal function is predominantly sustained by ATP production through glucose metabolism via oxidative phosphorylation. Ischemic stroke disrupts cerebral blood flow, leading to restricted glucose and oxygen supply, which in turn impairs normal energy metabolism [28]. A significant reduction in mitochondrial membrane potential and subsequent impairment of ATP production are key hallmarks of the initiation of apoptosis. Both IF and FC analysis (Figure 3A–D) demonstrated that OGD/R treatment resulted in a marked reduction in mitochondrial membrane potential in SH‐SY5Y cells. Notably, treatment with varying doses of Gas significantly attenuated this decrease in membrane potential. ATP levels in SH‐SY5Y cells were significantly decreased in the OGD/R group compared to the control group (Figure 3E). Notably, treatment with Gas effectively mitigated the reduction in ATP levels. NAD^+^ and NADH function as electron carriers in redox reactions, facilitating ATP synthesis. The balance between these two cofactors is crucial for maintaining normal energy metabolism [29, 30]. OGD/R significantly suppressed the production of NAD^+^ and NADH in SH‐SY5Y neuronal cells, leading to an imbalance between these key cofactors (Figure 3F–H). Treatment with Gas effectively mitigated these disruptions and restored energy metabolism in SH‐SY5Y cells.

### 3.4 Gas Mediates Mitochondrial Fusion Through Activation of the AMPK‐OPA1 Signaling Pathway to Protect SH‐SY5Y Cells After OGD/R

**FIGURE 4:**
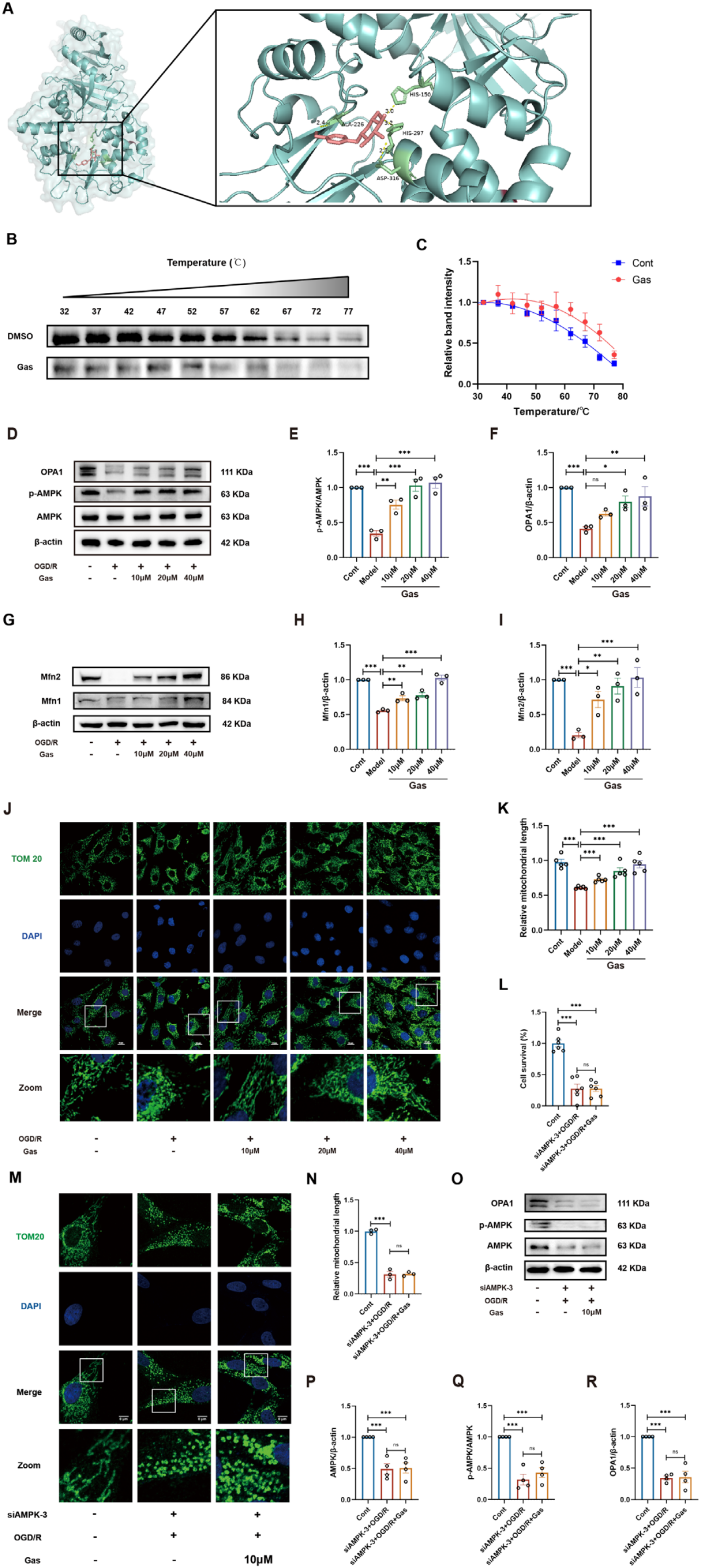
Gas mediates mitochondrial fusion through activation of the AMPK‐OPA1 signaling pathway to protect SH‐SY5Y cells after OGD/R. (A) Binding mode of Gas‐AMPK. (B) A CETSA was conducted to evaluate the binding of AMPK to Gas (*n* = 3). (C) The thermal stability of AMPK is quantified in the panel shown in (B), indicating an increase due to Ga binding, with a change in the melting temperature (ΔTm) of approximately 10°C. (D–F) WB analysis showing the expression of key proteins in the AMPK‐OPA1 signaling pathway in SH‐SY5Y cells following OGD/R and Gas intervention (*n* = 3). (G–I) Expression of Mfn1 and Mfn2 proteins in SH‐SY5Y cells following OGD/R and Gas intervention (*n* = 3). (J, K) Mitochondrial morphology of SH‐SY5Y cells after OGD/R and Gas intervention and quantitative analysis of average mitochondrial length (*n* = 5). (L) Cell viability of SH‐SY5Y cells after OGD/R and Gas intervention under AMPK silencing by CCK‐8 assay (*n* = 6). (M, N) Mitochondrial morphology of SH‐SY5Y cells after OGD/R and Gas intervention under AMPK silencing and quantitative analysis of average mitochondrial length (*n* = 3). (O–R) WB analysis showing the expression of key proteins in the AMPK‐OPA1 signaling pathway in SH‐SY5Y cells after OGD/R and Gas intervention under AMPK silencing (*n* = 4). Data are presented as mean ± SEM; *n* ≥ 3 independent experiments. *p < 0.05, **p < 0.01, ***p < 0.001.

Gas markedly improved mitochondrial function and energy metabolism in SH‐SY5Y cells subjected to OGD/R, suggesting its neuroprotective effects may involve modulation of the AMPK‐OPA1 signaling pathway. To explore whether Gas mediates neuroprotection through the AMPK‐OPA1 pathway, molecular docking simulations were conducted to assess its binding affinity to AMPK (Figure 4A). The results identified seven key amino acid residues within the AMPK binding site (HIS150, HIS297, ALA226, ASP316) that interact with Gas, indicating its potential to activate AMPK. A CETSA confirmed the molecular interaction between AMPK and Gas. The binding of Gas was found to increase the thermal stability of AMPK (Figure 4B) by approximately 10°C (Figure 4C), suggesting that Gas directly interacts with AMPK in the cellular environment. Furthermore, consistent with this hypothesis, Gas treatment significantly upregulated the AMPK‐OPA1 pathway in SH‐SY5Y cells following OGD/R compared to the model group (Figure 4D–F). Additionally, Gas treatment increased the expression of mitochondrial outer membrane fusion proteins Mfn1 and Mfn2 (Figure 4G–I). IF analysis further demonstrated that Gas promoted mitochondrial fusion in SH‐SY5Y cells, leading to an increase in mitochondrial length (Figure 4J,K).

To further investigate the relationship between Gas, AMPK‐OPA1 signaling, and mitochondrial morphological changes, we silenced AMPK expression. The experimental results (Figure 4O,P) demonstrated that AMPK silencing abolished the ability of Gas to activate the AMPK‐OPA1 pathway, thereby preventing mitochondrial fusion and its associated neuroprotective effects (Figure 4M,N). Under these conditions, mitochondria retained a donut‐shaped morphology. These findings indicate that AMPK is essential for the Gas‐mediated activation of OPA1, highlighting its critical role in regulating mitochondrial dynamics and neuroprotection.

### 3.5 Gas Ameliorates Cerebral Ischemia–Reperfusion Injury in MCAO/R Rats

**FIGURE 5:**
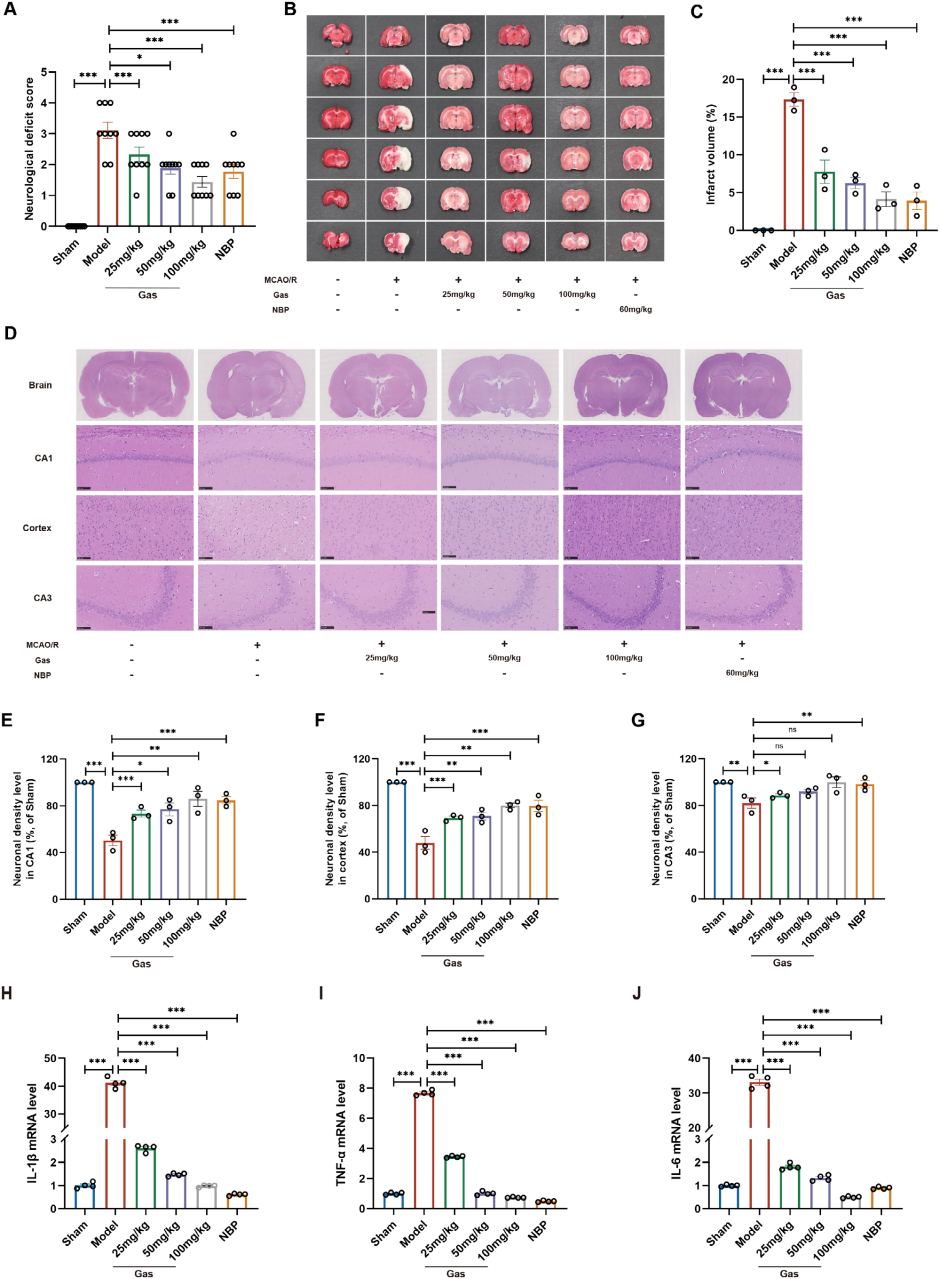
Gas ameliorates cerebral ischemia–reperfusion injury in MCAO/R rats. (A) Neurological scores of rats in each group (*n* = 9). (B, C) TTC staining of brain sections and quantitative analysis of brain infarct volume (*n* = 6). (D–G) Representative HE staining images and quantitative analysis of relative neuronal densities in the cortical and hippocampal CA1 and CA3 regions of MCAO/R rats treated with or without Gas (*n* = 3). HE staining illustrates morphological changes, while the quantitative analysis presents relative neuronal densities across groups. (H–J) RT‐PCR was performed to analyze the mRNA expression levels of pro‐inflammatory cytokines IL‐1β, TNF‐α, and IL‐6 in brain regions of MACO/R rats (*n* = 4). Data are presented as mean ± SEM; *n* ≥ 3 independent experiments. *p < 0.05, **p < 0.01, ***p < 0.001.

To assess the therapeutic potential of Gas in ischemic stroke, a rat MCAO/R model was established using the intraluminal filament method, involving 2 h of occlusion followed by 24 h of reperfusion. At 24 h post‐reperfusion, the Sham group exhibited normal neurological behavior, whereas the MCAO/R model group demonstrated significant neurological impairments, including left forelimb extension deficits, walking difficulties, circling to the left, and tipping, leading to markedly elevated neurological scores. Treatment with Gas or the positive control NBP significantly ameliorated these deficits, as reflected by improved left forelimb extension, enhanced walking flexibility, and overall mobility, along with a significant reduction in neurological scores (Figure 5A). Histological analysis showed no infarct lesions in the Sham group, while the MCAO/R model group displayed significant infarct volumes, with a distinct ischemic core and penumbra observed 24 h after ischemia (Figure 5B,C). Gas treatment significantly reduced infarct volumes compared to the MCAO/R model group, with the high‐dose Gas group demonstrating effects comparable to those of NBP. HE staining further revealed intact cellular morphology in the Sham group, characterized by clear nuclear outlines, prominent nucleoli, and the absence of tissue damage or edema (Figure 5D–G). In contrast, the MCAO/R model group exhibited severe neuronal damage, including enlarged neuronal gaps, pronounced edema, reduced cell density, irregular morphology, nuclear pyknosis, and cytoplasmic vacuolization. Gas treatment significantly alleviated these pathological changes by reducing edema and neuronal gaps, restoring cellular morphology, increasing cell density, and minimizing nuclear pyknosis and cytoplasmic vacuolization. To evaluate the anti‐inflammatory effects of Gas, RT‐PCR analysis was performed to measure the expression of pro‐inflammatory factors TNF‐α, IL‐6, and IL‐1β in brain tissues. Compared to the MCAO/R model group, Gas treatment significantly suppressed the expression of these inflammatory markers (Figure 5H–J). These results collectively demonstrate that Gas effectively protects against neuronal damage and inflammation induced by cerebral ischemia–reperfusion injury.

### 3.6 Gas Ameliorates Nissl Bodies Abnormality and Modulates Astrocytic Activation in MCAO/R Rats

**FIGURE 6:**
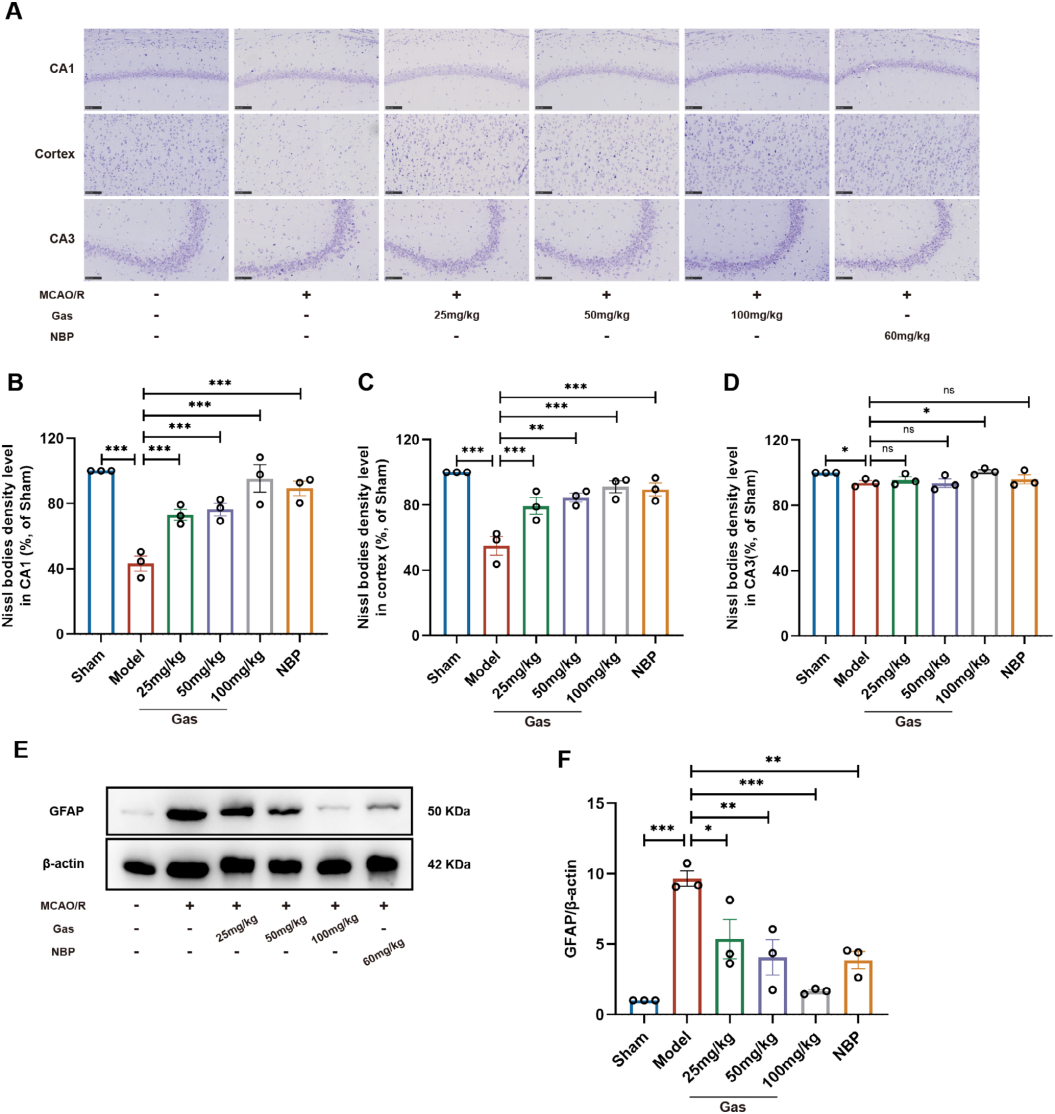
Gas activation of the AMPK‐OPA1 signaling pathway to promote mitochondrial fusion attenuates neurological injury in MCAO/R rats (*n* = 3). (A–D) Representative Nissl staining and quantitative analysis of Nissl body density in cortical and hippocampal regions of MCAO/R rats treated with or without Gas (*n* = 3). (E, F) WB analysis was performed to evaluate the effect of Gas on the expression of GFAP in the brain tissues of MCAO/R rats. Data are presented as mean ± SEM; *n* = 3 independent experiments. *p < 0.05, **p < 0.01, ***p < 0.001.

Nissl staining was performed to assess changes in Nissl bodies within the brain tissue of rats (Figure 6A–D). In the Sham group, neuronal cells displayed normal morphology, with abundant neurons and Nissl bodies that were densely and orderly distributed. The cytoplasm was rich, and the cell membranes, nuclear membranes, and nucleoli were well‐defined, with no apparent abnormalities. In contrast, the MCAO/R group showed severe pathological changes, including fragmented Nissl bodies, prominent edema, necrosis, and marked vacuolization. Neuronal cells were sparse and disorganized, with enlarged perinuclear spaces around Nissl bodies. Additionally, neurons exhibited swelling or shrinkage; nucleoli were absent, nuclear membranes were dissolved, and nuclei exhibited pyknosis. Gas treatment significantly mitigated ischemia–reperfusion‐induced damage compared to the MCAO/R group. Vacuolization was reduced, and abnormalities in neurons and Nissl bodies were limited to a few cells. Most neurons and Nissl bodies appeared normal, with an orderly arrangement. Neuronal density was notably increased, and cytoplasmic shrinkage was alleviated. During cerebral ischemia, astrocytes are among the first cells to sustain ischemic damage. Glial fibrillary acidic protein (GFAP), a type III intermediate filament protein and a major component of the astrocytic cytoskeleton, is widely used as a biomarker for assessing astrocyte activation. Changes in GFAP expression serve as an indicator of the extent of pathological damage in brain tissue following stroke. In the MCAO/R model (Figure 6E,F), GFAP expression was significantly upregulated, indicating pronounced astrocyte activation. Treatment with Gas effectively suppressed the excessive activation of GFAP, suggesting that Gas can modulate astrocytic activation and mitigate astrocyte‐mediated responses following cerebral ischemia.

### 3.7 Gas Activation of the AMPK‐OPA1 Signaling Pathway to Promote Mitochondrial Fusion Attenuates Neurological Injury in MCAO/R Rats

**FIGURE 7:**
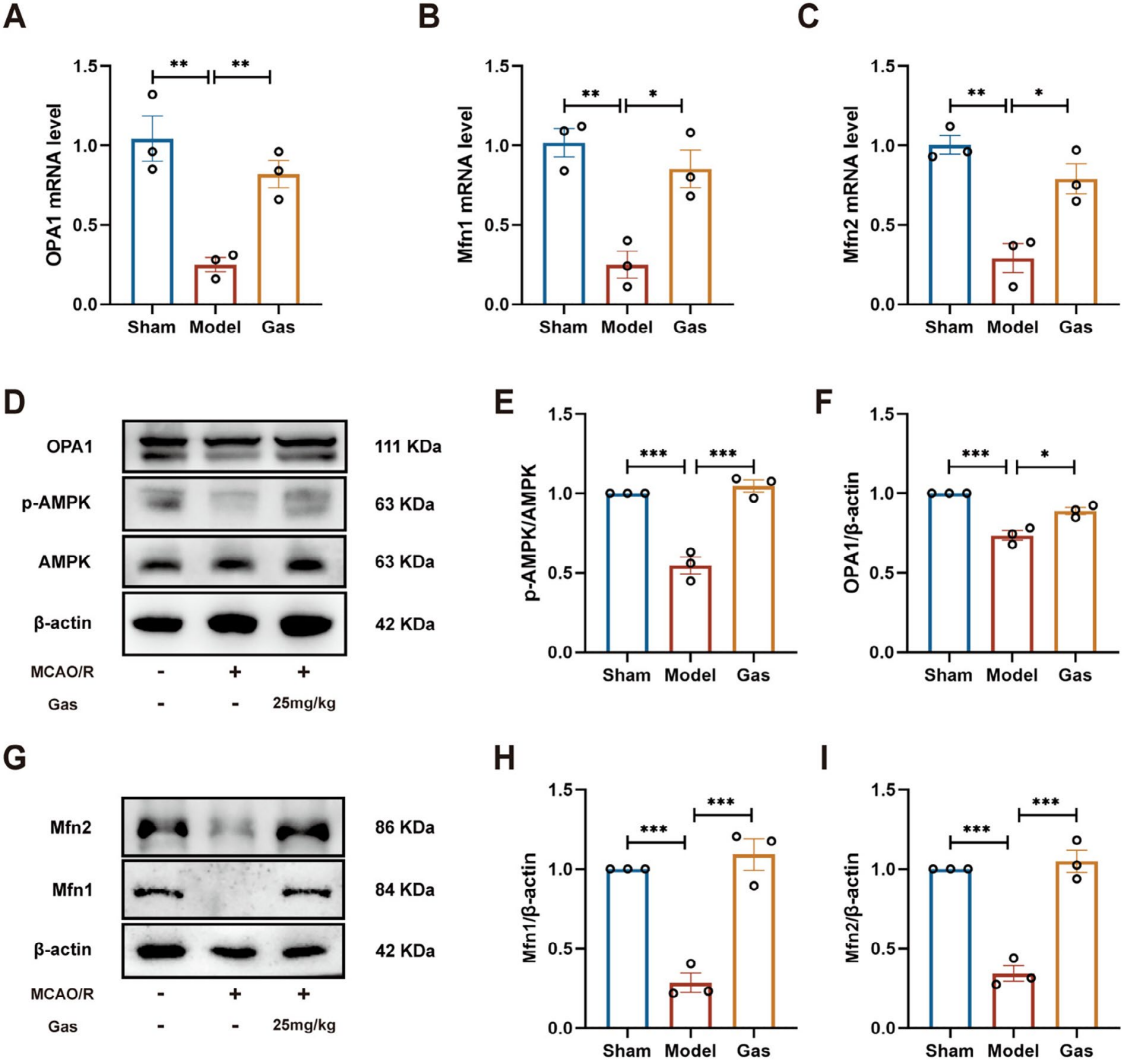
Gas activation of the AMPK‐OPA1 signaling pathway to promote mitochondrial fusion attenuates neurological injury in MCAO/R rats. (A–C) RT‐PCR analysis of mRNA expression levels of OPA1, Mfn1, and Mfn2 in the brain tissues of MCAO/R rats (*n* = 3). (D–F) WB analysis was conducted to assess the effect of Gas on the expression levels of proteins AMPK and OPA1 in the brain tissues of MCAO/R rats (*n* = 3). (G–I) WB analysis was conducted to assess the effect of Gas on the expression levels of mitochondrial fusion proteins Mfn1 and Mfn2 in the brain tissues of MCAO/R rats (*n* = 3). Data are presented as mean ± SEM; *n* = 3 independent experiments. *p < 0.05, **p < 0.01, ***p < 0.001.

We assessed the neuroprotective effect of Gas on MCAO/R rats by measuring the expression levels of mitochondrial fusion‐related mRNAs (OPA1, Mfn1, and Mfn2) through RT‐PCR. RT‐PCR analysis demonstrated that Gas treatment markedly increased the expression of OPA1, Mfn1, and Mfn2 mRNAs in the brain tissue of MCAO/R rats (Figure 7A–C). We further investigated whether Gas exerts neuroprotection in MCAO/R rats through the AMPK‐OPA1 pathway by conducting Western blot analysis. Notably, the experimental results confirmed this hypothesis. WB analysis revealed that the AMPK‐OPA1 signaling pathway was significantly suppressed in the brain tissue of MCAO/R rats (Figure 7D–F). However, Gas treatment effectively activated this pathway. Additionally, we assessed the impact of Gas on the expression of mitochondrial fusion proteins Mfn1 and Mfn2 in the ischemic brain regions of MCAO/R rats. Compared to the Sham group, the MCAO/R model group exhibited a significant reduction in the expression of both Mfn1 and Mfn2 (Figure 7G–I). In contrast, Gas administration notably restored the expression levels of Mfn1 and Mfn2 in the ischemic‐reperfused brain regions compared to the MCAO/R group.

## 4 Discussion

Among non‐communicable diseases, stroke is the second leading cause of death and the third cause of disability in the world [1]. The pathogenesis of ischemic stroke involves a complex series of physiological and molecular events, primarily driven by reduced blood flow, which leads to impaired energy metabolism, dysregulation of cellular ion homeostasis, oxidative stress, and inflammatory responses [31]. In the current study, we provided evidence to explain the core role played by OPA1‐related mitochondrial protection in the pathogenesis of cerebral I/R injury. Moreover, we found that Gas supplementation could attenuate cerebral I/R injury and relieve mitochondrial stress by activating OPA1‐related mitochondrial fusion in a manner dependent on the AMPK signaling pathway. To our knowledge, this study is the first investigation to explore the role of Gas‐modified mitochondrial fusion in the setting of cerebral I/R injury. These findings have substantiated the sufficiency of OPA1‐related mitochondrial fusion to mitigate cerebral reperfusion damage, as well as the necessity of Gas to activate OPA1‐induced mitochondrial protective machinery.

Mitochondria are pivotal organelles central to cellular energy production, oxidative stress regulation, and calcium homeostasis. The dynamic balance between mitochondrial fusion and fission is essential for maintaining mitochondrial integrity and enabling cellular adaptation to intra‐ and extracellular stressors [32]. Mitochondrial fusion is mediated by the outer membrane GTPase proteins Mfn1 and Mfn2, along with the inner membrane protein OPA1. Fission, by contrast, is predominantly orchestrated by DRP1, which interacts with outer membrane receptors such as mitochondrial fission factor (MFF) and MID49/51. These processes are tightly regulated by post‐translational modifications, including phosphorylation and acetylation, as well as by mitochondrial membrane potential, calcium signaling, and oxidative stress. Mitochondrial fission is an early and critical event in stress responses, often preceding apoptosis. DRP1, a key modulator of mitochondrial dynamics, undergoes phosphorylation, facilitating its recruitment to the outer mitochondrial membrane. At these sites, DRP1 assembles into oligomeric complexes that drive membrane scission, resulting in mitochondrial fragmentation. This fragmentation process increases mitochondrial outer membrane permeability (MOMP), promoting the release of pro‐apoptotic factors such as cytochrome c into the cytosol, thereby initiating the apoptotic cascade [33]. Conversely, mitochondrial hyperfusion acts as an adaptive mechanism to counter cellular stress and preserve viability. This state of extended mitochondrial network connectivity supports the redistribution of functional mitochondrial components, enhances bioenergetic efficiency, and mitigates oxidative damage. AMPK, a key serine/threonine kinase, is ubiquitously expressed in various cells and tissues. As a primary cellular energy sensor, AMPK is activated under conditions of low energy [13]. It functions by phosphorylating specific enzymes and regulatory proteins to enhance ATP production and suppress ATP consumption. However, the importance of mitochondrial dynamics in cerebral I/R injury has not been recognized. In the present study, we found that OPA1 expression was significantly suppressed in SH‐SY5Y cells after OGD/R by WB experiments, which demonstrated the importance of the AMPK‐OPA1 pathway in the pathogenesis of stroke.

Moreover, despite the extensive research that has been conducted to explain the neuroprotective effects of Gas [34, 35, 36], no study has been performed to confirm the influence of Gas on mitochondrial fusion. Our results provided answers for these questions. First, we demonstrated that Gas markedly improved mitochondrial function and energy metabolism in SH‐SY5Y cells subjected to OGD/R, suggesting its neuroprotective effects may involve modulation of the AMPK‐OPA1 signaling pathway. Second, molecular docking simulations were conducted to assess its binding affinity to AMPK. The results identified seven key amino acid residues within the AMPK binding site (HIS150, HIS297, ALA226, ASP316) that interact with Gas, indicating its potential to activate AMPK. Third, to further investigate the relationship between Gas, AMPK‐OPA1 signaling, and mitochondrial morphological changes, we silenced AMPK expression. The experimental results demonstrated that AMPK silencing abolished the ability of Gas to activate the AMPK‐OPA1 pathway, thereby preventing mitochondrial fusion and its associated neuroprotective effects.

In vivo study, a rat MCAO/R model was established using the intraluminal filament method; Gas treatment significantly alleviated these pathological changes by reducing edema and neuronal gaps, restoring cellular morphology, increasing cell density, and minimizing nuclear pyknosis and cytoplasmic vacuolization compared to the MCAO/R model group. WB analysis revealed that Gas activation of the AMPK‐OPA1 signaling pathway to promote mitochondrial fusion attenuates neurological injury in MCAO/R rats.

There are several limitations in the present study. First, it seems to be better to use OPA1^fl/fl^ mice as a control group to exclude many background differences. Second, AMPK has been identified as the upstream mediator of OPA1, and it remains unknown whether OPA1 has some feedback to the AMPK pathway. Our in vitro experiments relied on a single cell type (e.g., SH‐SY5Y cell line), which may not fully recapitulate the complex cellular interactions occurring in the ischemic brain. Second, in our in vivo experiments, we focused exclusively on GFAP‐labeled astrocyte activation without evaluating the contributions of other critical cell types (e.g., microglia, neurons, or endothelial cells) to the pathophysiology of ischemic stroke. Despite the aforementioned methodological limitations of this study, we have systematically elucidated for the first time the molecular mechanism by which tianphenin exerts its neuroprotective effects through the regulation of AMPK‐OPA1. These findings provide a new experimental basis for understanding the therapeutic potential of active ingredients in traditional Chinese medicine.

## 5 Conclusion

This study elucidates the neuroprotective effects of Gas in mitigating cerebral I/R injury, primarily through activation of the AMPK‐OPA1 signaling pathway to regulate mitochondrial dynamics. Gas demonstrated significant protective effects in both in vitro and in vivo models by suppressing inflammatory responses, preserving mitochondrial membrane potential, promoting mitochondrial fusion, and improving energy metabolism. By modulating mitochondrial fusion states, Gas effectively attenuated neurological deficits and tissue damage in stroke models. These findings provide robust support for the development of Gas‐based therapeutic strategies for stroke and deepen our understanding of the pathophysiological mechanisms underlying ischemic stroke.

## Author Contributions

**Zihan Liu:** investigation, methodology, visualization, writing – original draft. **Zeyu Han:** formal analysis, validation, methodology, writing – original draft. **Wenshuai Bao:** resources, writing – review and editing. **Yihan Guo:** software, writing – review and editing. **Yuan Yuan:** data curation, writing – review and editing. **Jianming Cheng:** conceptualization, supervision. **Jie Zhang:** project administration, writing – review and editing. **Yang Hu:** conceptualization, funding acquisition, writing – review and editing.

## Conflicts of Interest

The authors declare no conflicts of interest.

## Supporting information


**Data S1:** cns70559‐sup‐0001‐DataS1.pdf.

**Table S1:** cns70559‐sup‐0002‐TableS1.docx.

## Data Availability

The data that support the findings of this study are available from the corresponding author upon reasonable request.
