# Supplementary material for: Gastrodin Attenuates Cerebral Ischemia–Reperfusion Injury by Enhancing Mitochondrial Fusion and Activating the AMPK‐OPA1 Signaling Pathway
Source: CNS Neurosci Ther. 2025 Aug 11;31(8):e70559. doi: 10.1111/cns.70559 (PMC12339906; doi:10.1111/cns.70559)

FIGURE 1 A Expression of AMPK and OPA1 in SH-SY5Y cells transfected with different AMPK siRNAs (n=3).

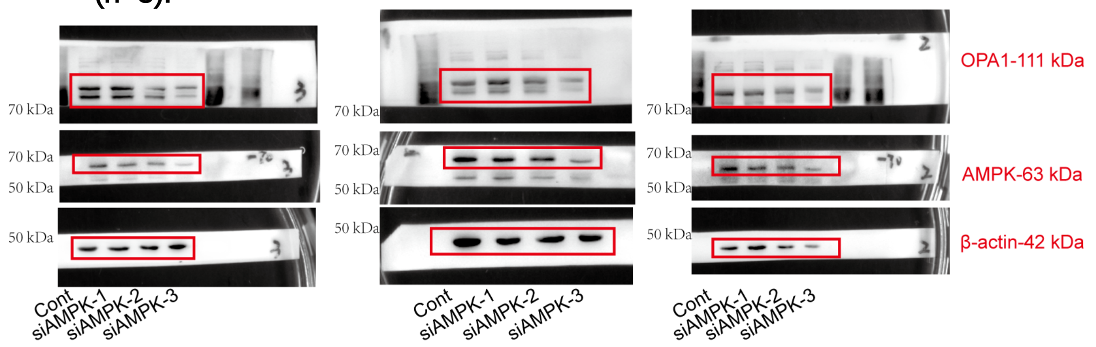

FIGURE 1E Expression of AMPK, p-AMPK, and OPA1 in SH-SY5Y cells following OGD/R or AMPK silencing (n=3).

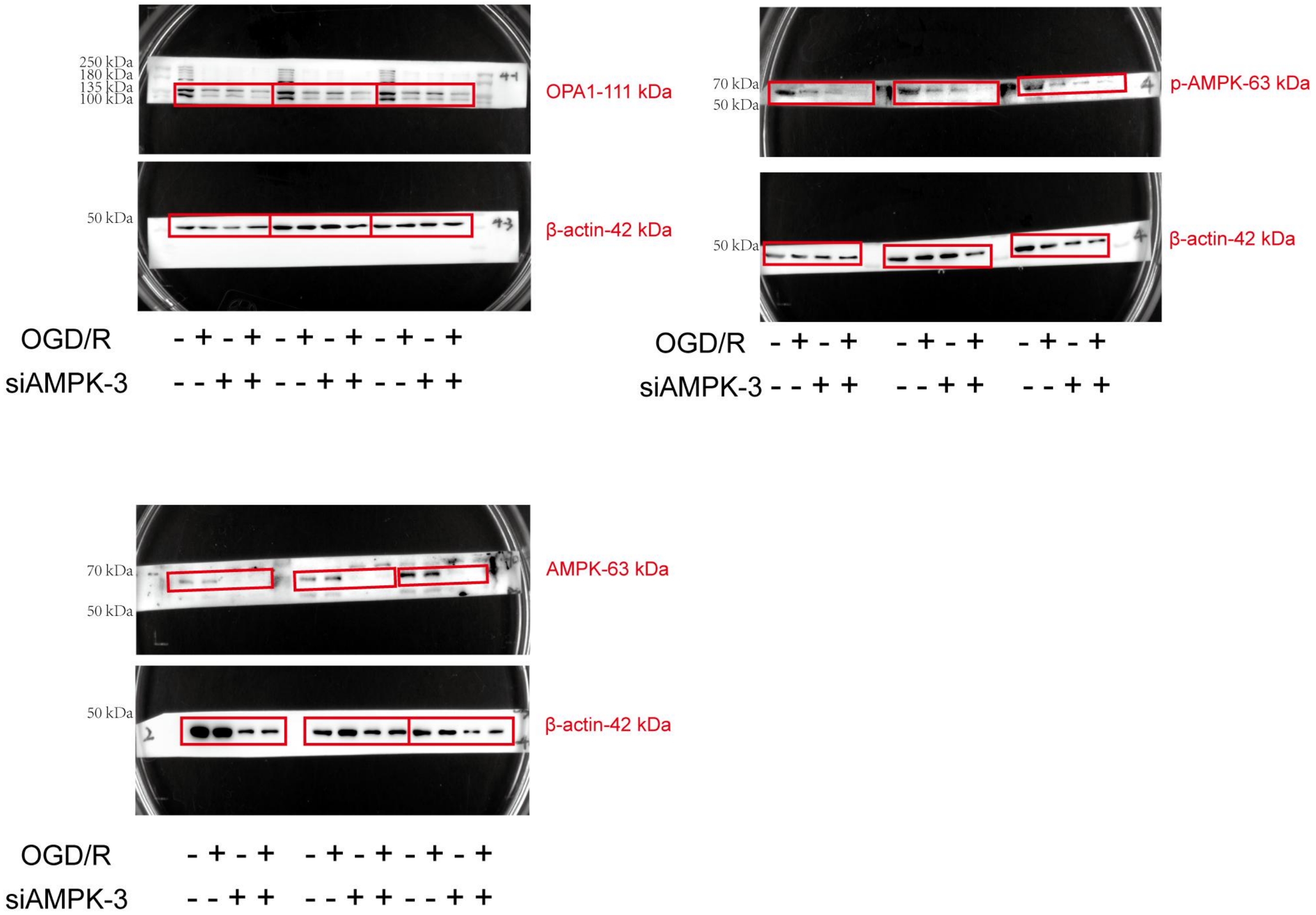

FIGURE 4B A CETSA was conducted to evaluate the binding of AMPK to Gas (n=3).

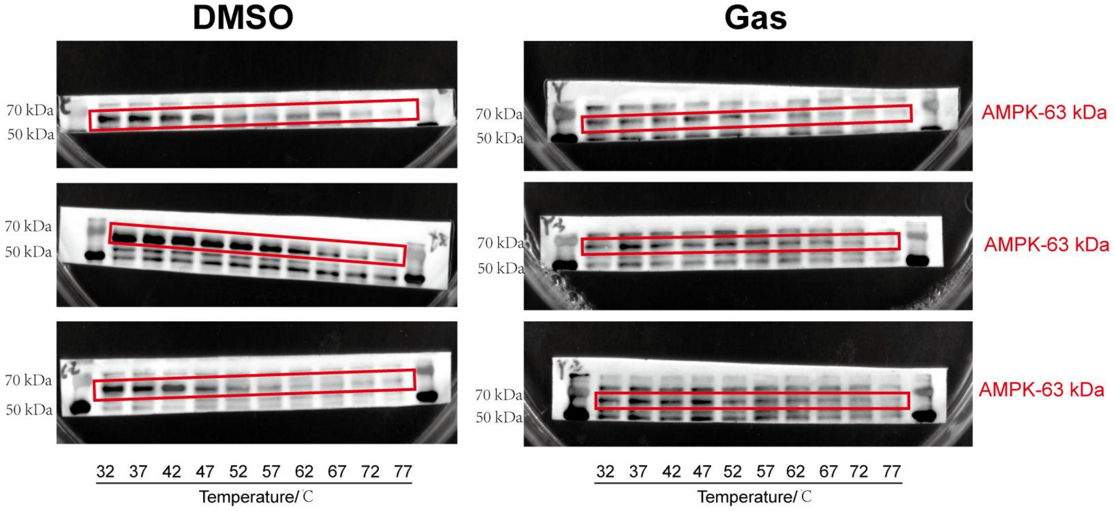

FIGURE 4D Expression of key proteins in the AMPK-OPA1 signaling pathway in SH-SY5Y cells following OGD/R and Gas intervention(n=3).

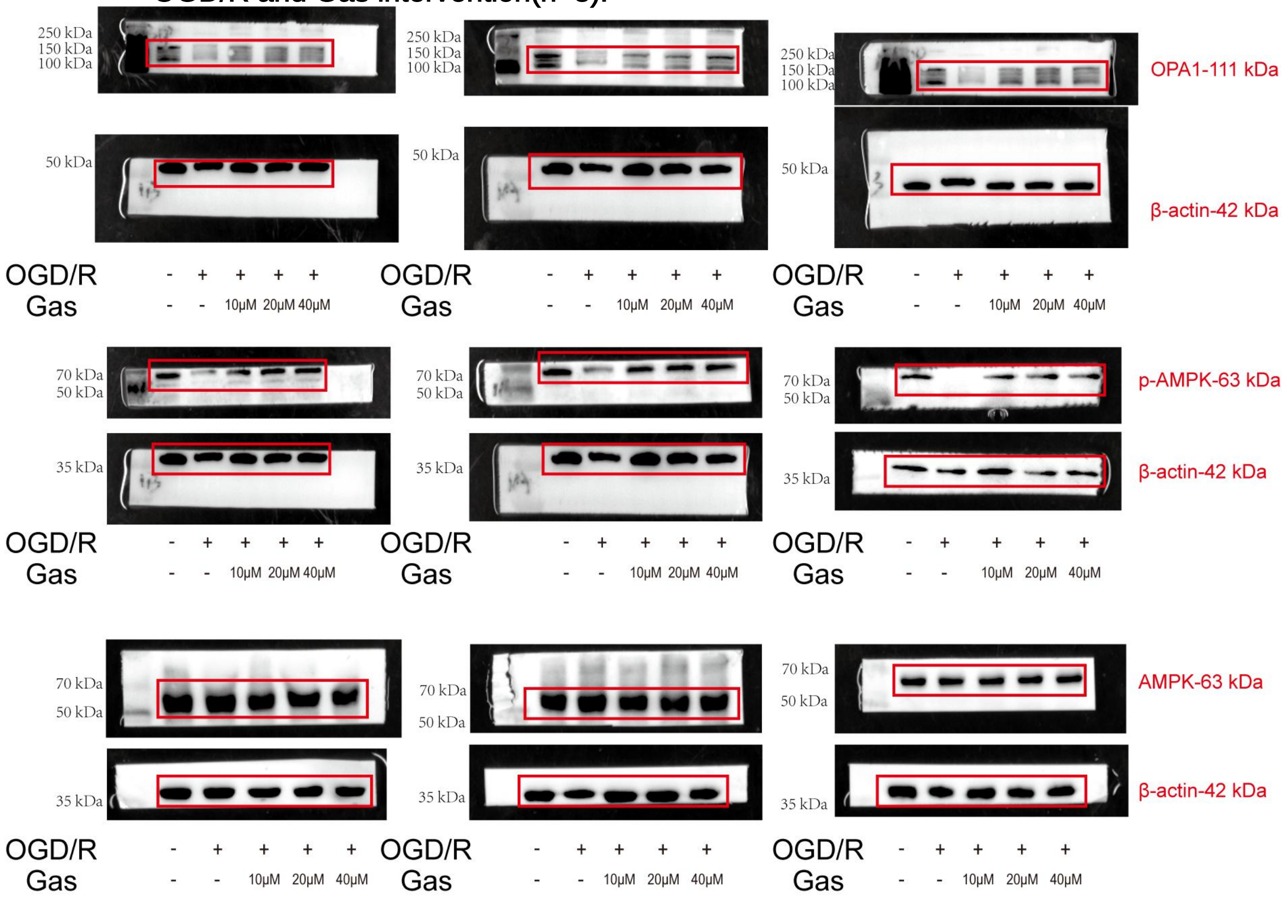

FIGURE 4G Expression of Mfn1 and Mfn2 proteins in SH-SY5Y cells following OGD/R and Gas intervention (n=3).

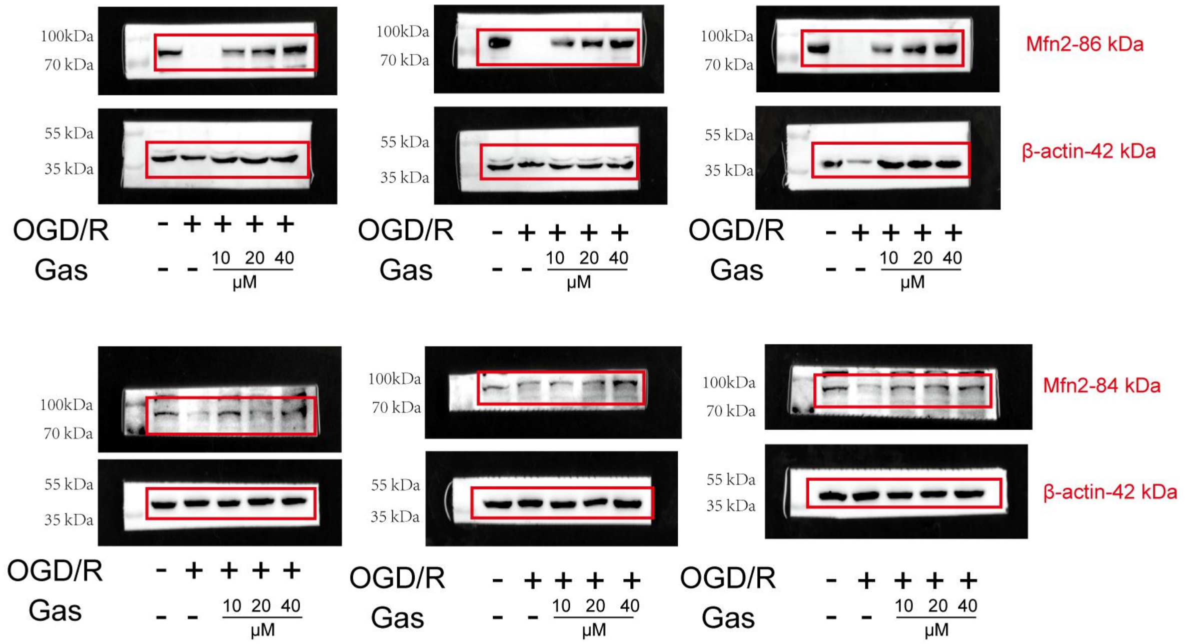

FIGURE 4O Expression of key proteins in the AMPK-OPA1 signaling pathway in SH-SY5Y cells after OGD/R and Gas intervention under AMPK silencing (n=4).

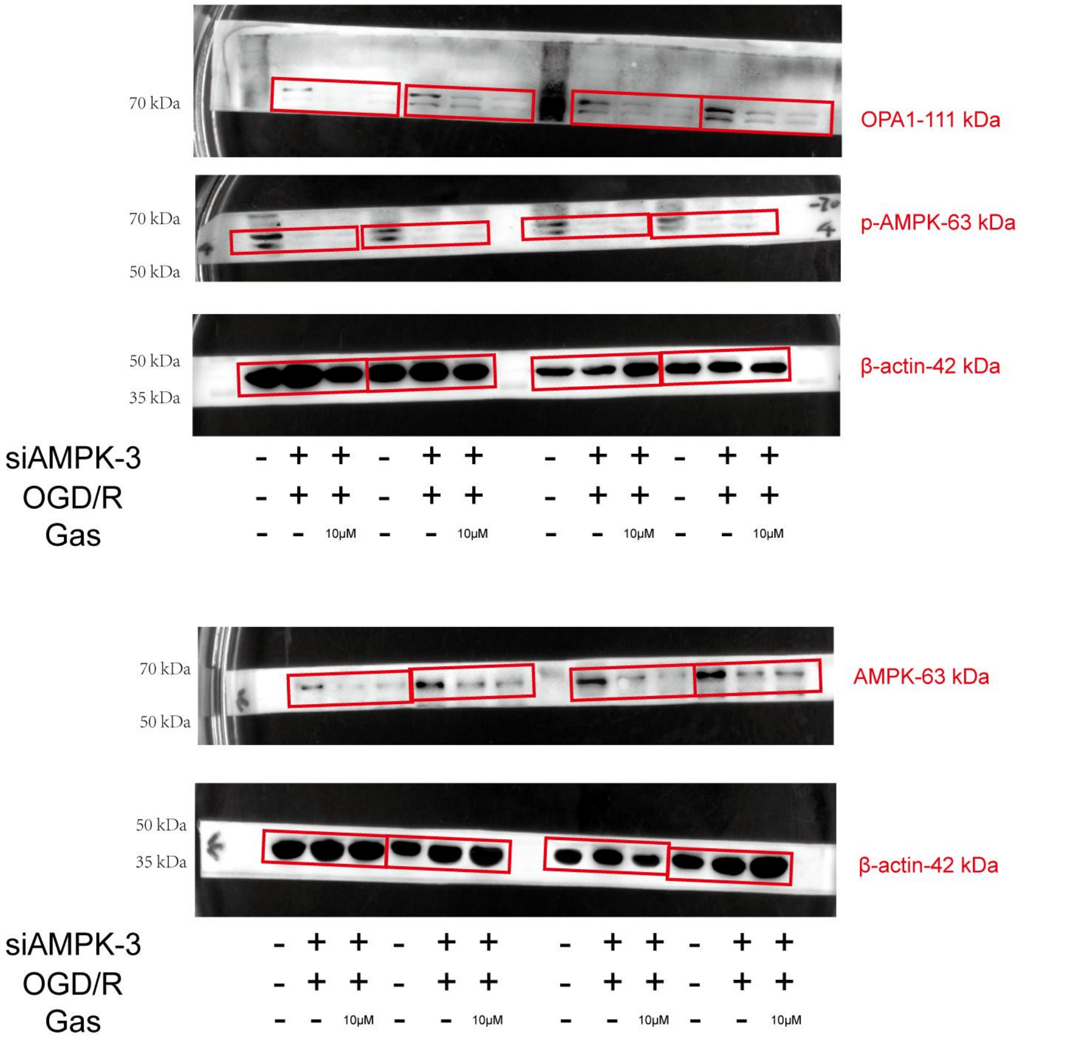

FIGURE 6E The effect of Gas on the expression of GFAP in the brain tissues of MCAO/R rats.

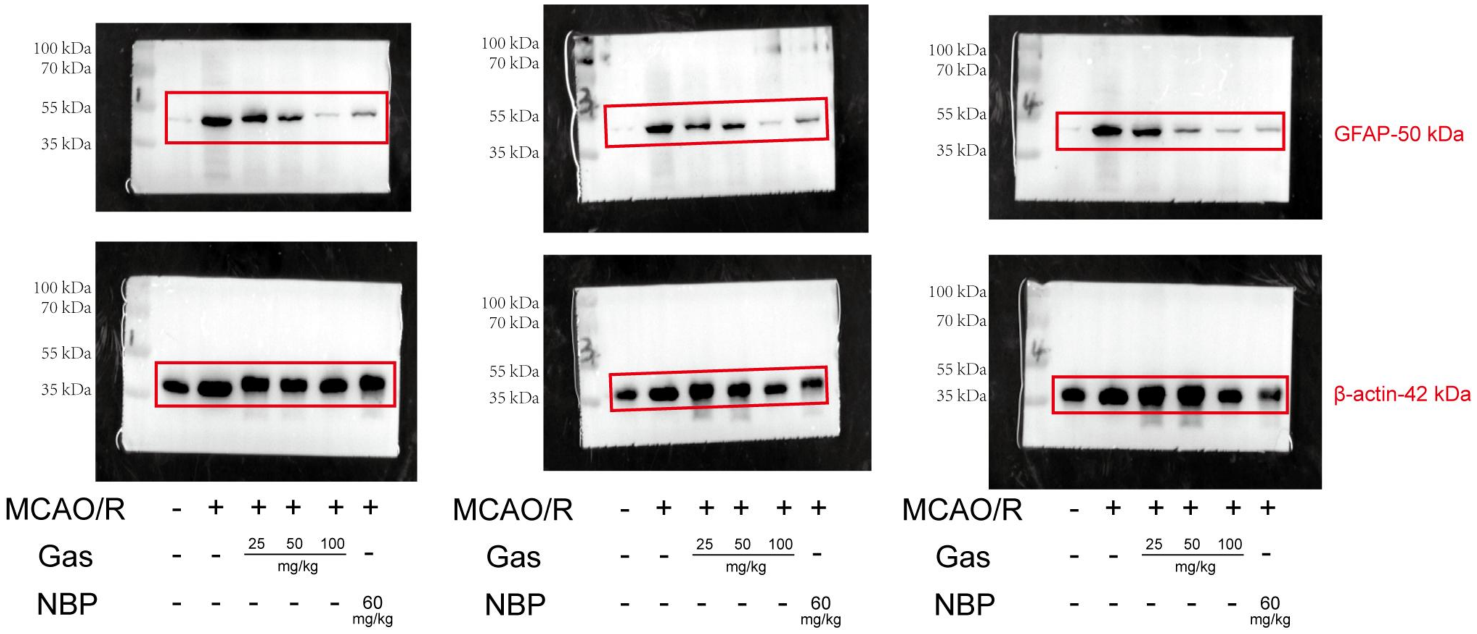

FIGURE 7D The effect of Gas on the expression levels of proteins AMPK and OPA1 in the brain tissues of MCAO/R rats (n=3).

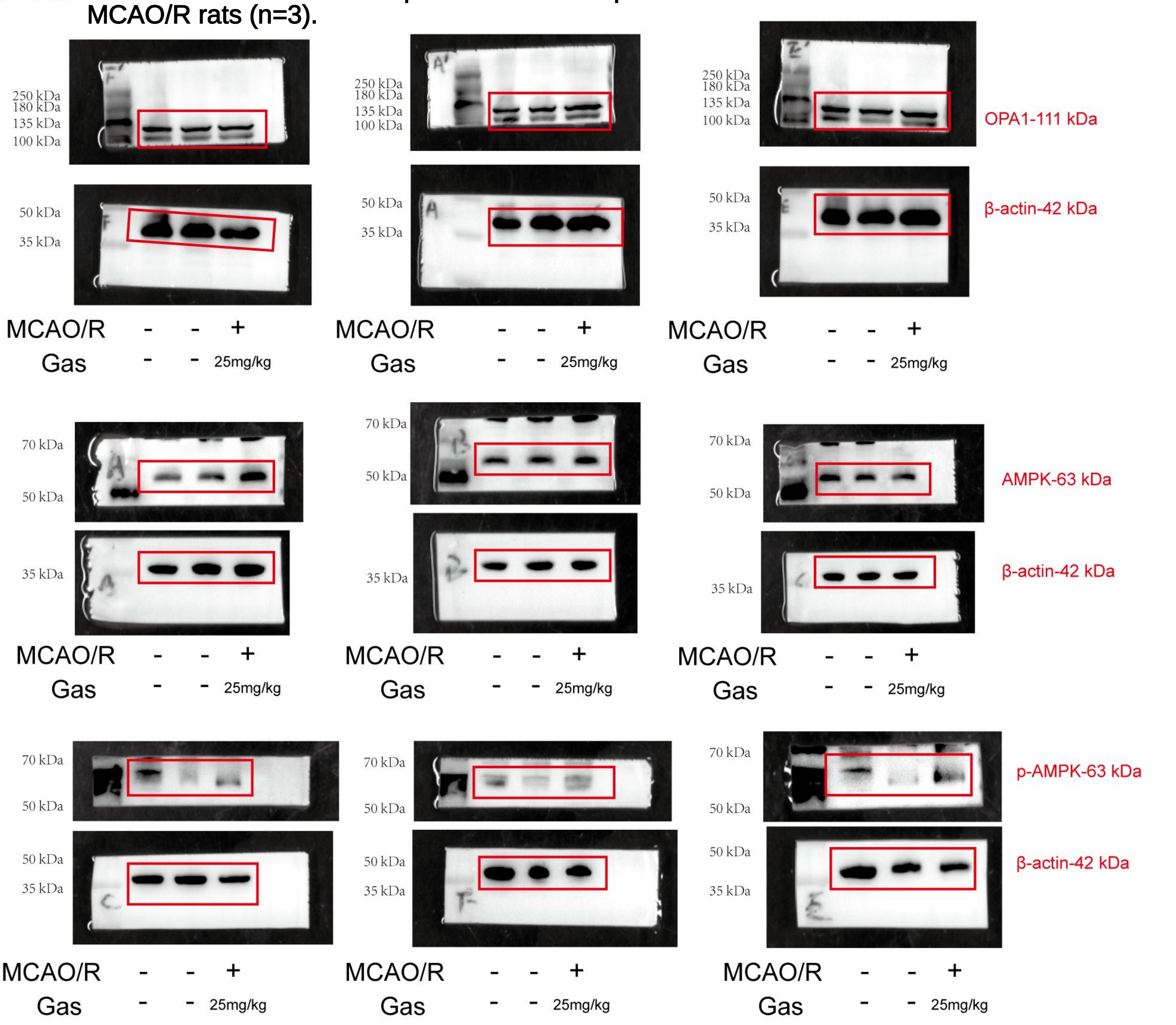

FIGURE 7G The effect of Gas on the expression levels of mitochondrial fusion proteins Mfn1 and Mfn2 in the brain tissues of MCAO/R rats (n=3).

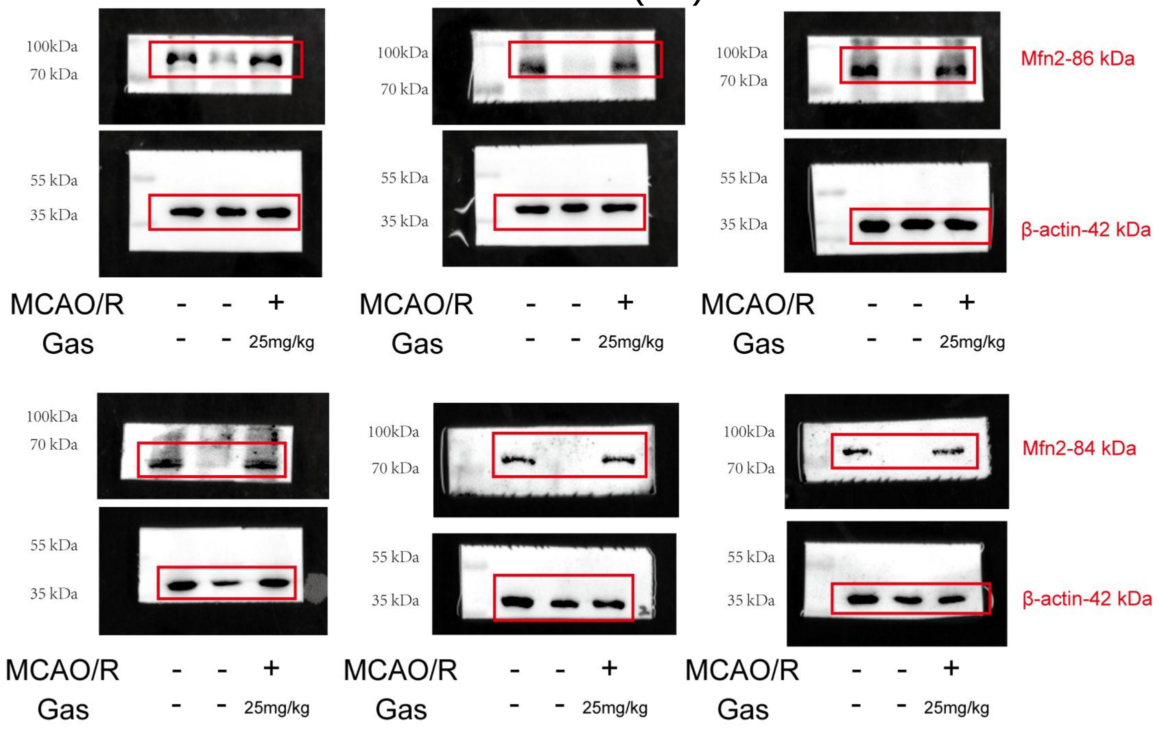

Supplement: Supplementary file 1 — Data S1: cns70559‐sup‐0001‐DataS1.pdf. [file CNS-31-e70559-s002.pdf]
