# Supplementary material for: Gastrodin Attenuates Cerebral Ischemia–Reperfusion Injury by Enhancing Mitochondrial Fusion and Activating the AMPK‐OPA1 Signaling Pathway
Source: CNS Neurosci Ther. 2025 Aug 11;31(8):e70559. doi: 10.1111/cns.70559 (PMC12339906; doi:10.1111/cns.70559)
Supplement: Supplementary file 2 — Table S1: cns70559‐sup‐0002‐TableS1.docx. [file CNS-31-e70559-s001.docx]

**Additional file: Table S1**

**Table S1.** Primer sequences for RT-PCR analysis

| **Species** | **Gene** | **Forward** | **Reverse** |
| --- | --- | --- | --- |
| Human | Actin | CATGTACGTTGCTATCCAGGC | CTCCTTAATGTCACGCACGAT |
| Human | IL-1β | ATGATGGCTTATTACAGTGGCAA | GTCGGAGATTCGTAGCTGGA |
| Human | TNF-a | CCTCTCTCTAATCAGCCCTCTG | GAGGACCTGGGAGTAGATGAG |
| Human | IL-6 | ACTCACCTCTTCAGAACGAATTG | CCATCTTTGGAAGGTTCAGGTTG |
| Mouse | IL-1β | CTTCTGGGCCTGCTGTTCA | CCAGCCTACTCATTGGGATCA |
| Mouse | TNF-a | CAGGCGGTGCCTATGTCTC | CGATCACCCCGAAGTTCAGTAG |
| Mouse | IL-6 | CTGCAAGAGACTTCCATCCAG | AGTGGTATAGACAGGTCTGTTGG |
| Mouse | IL-1β | GAAATGCCACCTTTTGACAGTG | TGGATGCTCTCATCAGGACAG |
| Mouse | OPA1 | TGGAAAATGGTTCGAGAGTCAG | CATTCCGTCTCTAGGTTAAAGCG |
| Mouse | Mfn1 | ATGGCAGAAACGGTATCTCCA | GCCCTCAGTAACAAACTCCAGT |
| Mouse | Mfn2 | AGAACTGGACCCGGTTACCA | CACTTCGCTGATACCCCTGA |
